# Positive p53 Expression Is Associated with Primary Endocrine Therapy Resistance in Locally Advanced Stage Luminal B HER2-Negative Breast Cancer Patients: A Cross-Sectional Study in Indonesia

**DOI:** 10.3390/diagnostics13111838

**Published:** 2023-05-24

**Authors:** Freda Halim, Yohana Azhar, Suwarman Suwarman, Eka Julianta Wahjoepramono, Bethy Hernowo

**Affiliations:** 1 Faculty of Medicine, Universitas Padjadjaran, Bandung 40161, West Java, Indonesia; r.yohana@unpad.ac.id; 2 Department of Surgery, Faculty of Medicine, Pelita Harapan University, Tangerang 15811, Banten, Indonesia; 3 Department of Surgery, Oncology, Head and Neck Division, Hasan Sadikin Hospital, Bandung 40161, West Java, Indonesia; 4 Department of Anesthesiology and Intensive Care, Universitas Padjadjaran, Bandung 40161, West Java, Indonesia; suwarman@unpad.ac.id; 5 Department of Neurosurgery, Faculty of Medicine, Pelita Harapan University, Tangerang 15811, Banten, Indonesia; eka.wahjoepramono@uph.edu; 6 Department of Anatomical Pathology, Universitas Padjadjaran, Bandung 40161, West Java, Indonesia; bethy.suryawathy@unpad.ac.id

**Keywords:** luminal B HER-2 negative, endocrine therapy resistance, p53, cross-sectional study

## Abstract

Luminal B HER2-negative breast cancer (BC) is the most common type in Indonesian BC patients, and frequently manifests with locally advanced staging. Recurrence often occurs within two years of the endocrine therapy course (primary endocrine therapy (ET) resistance). p53 mutation often exists in luminal B HER2-negative BC, but its application as an ET resistance predictor in those populations is still limited. The primary purpose of this research is to evaluate p53 expression and its association with primary ET resistance in luminal B HER2-negative BC. This cross-sectional study compiled 67 luminal B HER2-negative patients’ clinical data during their pre-treatment period until they completed a two-year course of endocrine therapy. They were divided into two groups: 29 patients with primary ET resistance and 38 without primary ET resistance. Pre-treatment paraffin blocks from each patient were retrieved, and the p53 expression difference between the two groups was analyzed. Positive p53 expression was significantly higher in patients with primary ET resistance [odds ratio (OR) of 11.78 (95% CI: 3.72–37.37, *p*-value < 0.0001)]. We conclude that p53 expression could be a beneficial marker for primary ET resistance in locally advanced luminal B HER2-negative BC.

## 1. Introduction

Breast cancer (BC) is the number one leading cancer case in Indonesia, according to GLOBOCAN 2020 data, both in incidence and mortality rate [1]. From several observations, luminal B, especially HER2-negative BC, is the most common type of BC in Indonesia, and most of the patients came with already locally advanced disease [2,3,4,5]. This BC type has a higher disease recurrence rate and higher mortality, resulting in a worse overall prognosis than luminal A BC, especially in advanced-stage disease [6,7,8].

The long-term prognosis of luminal B BC depends on the success of endocrine therapy (ET), in which the patients will receive suppression of estrogen production and/or estrogen receptor (ER) blockade or ER degradation for years [9]. Nevertheless, most of the disease recurrence of luminal B BC occurs within two years of the ET period, causing a distinct pattern of higher recurrence during 2–5 years of treatment compared to the non-luminal patients’ group [7]. Such recurrence and/or disease progressiveness occurs within the first two years of ET, referred to as primary ET resistance [10]. The primary ET resistance will lead to the loss of ET as a primary and long-term modality to reduce recurrence and mortality in luminal B BC patients, resulting in a poor prognosis in this patient group [11].

Although ET resistance has been a paramount problem for patients with HR (+) BC and clinicians, no established biological marker related to ET resistance occurrence has been distinguished [12]. A biological marker will undoubtedly benefit patients and clinicians in providing prognostic assessment and understanding the importance of careful monitoring [13,14].

p53 has been studied in many studies for its role in luminal BC [15,16,17,18]. Its expression is often found in luminal B, especially luminal B HER2-negative BC, about 44–58% [19,20]. But to our knowledge, its role in primary ET resistance, specifically in luminal B HER2 negatives, has never been studied before.

The primary purpose of this research is to evaluate p53 expression and its association with primary ET resistance in luminal B HER2-negative patients. Our main hypothesis is that p53 protein expression, as a surrogate marker for p53 mutations, will be expressed higher in patients with primary ET resistance than in the group without primary ET resistance.

## 2. Materials and Methods

### 2.1. Patients and Samples

This research is a retrospective cross-sectional study conducted from April to November 2022 in four hospitals located in three provinces in Indonesia: Hasan Sadikin General Hospital and Santosa General Hospital in Bandung City, West Java Province; Siloam Hospital Karawaci in Tangerang City, Banten Province; and Mochtar Riady Comprehensive Cancer Center (MRCCC) Siloam Hospital Semanggi in the Special Region of Jakarta, Indonesia’s capital city.

The study protocol was approved by the Institutional Review Board of Padjajaran University (ethical registration number 2112061306) on 14 March 2022. All ethical review boards reviewed and agreed to the study protocol. We followed the Strengthening of Reporting Observational Studies in Epidemiology (STROBE) guidelines [21].

We collected data from the Pathology Anatomy Laboratory and Medical Records in those four hospitals for newly diagnosed locally advanced stage (IIIA–IIIC) luminal B HER2-negative patients from 2016–2022; all patients were treated according to the Indonesian guidelines for breast cancer treatment published by the Indonesian Health Ministry in 2018 [22].

All patients underwent the pre-treatment biopsy procedure (open or core needle biopsy) and neoadjuvant chemotherapy. Radiotherapy was not mandatory; it was only accomplished if indicated. All patients underwent a mastectomy procedure (simple or modified radical mastectomy) and received ET for five years or more. However, all patients included in this research must complete at least two years of ET.

The inclusion criteria for our study were locally advanced (stages IIIA–IIIC) luminal B HER2-negative BC, complete clinical data, and acceptable quality of paraffin block to be tested. The exclusion criteria for our study were obesity (BMI ≥ 30 kg/m^2^), bilateral breast cancer, metastatic disease when pre-treatment staging was performed, being unresponsive to neoadjuvant chemotherapy, and patients with any missing data. Confounding variables in the study were the patient’s age, stage and histological grade, ER expression level, chemotherapy, and radiotherapy usage.

Further, all patients were divided into two groups, i.e., groups with and without primary ET. We defined primary ET resistance as any clinical recurrence or disease progressiveness within 2 years of ET course [10]. Then we compared the two groups for their clinicopathological characteristics, as depicted in Table 1.

### 2.2. Immunohistochemical Analysis for p53 Expression

**Figure 1:**
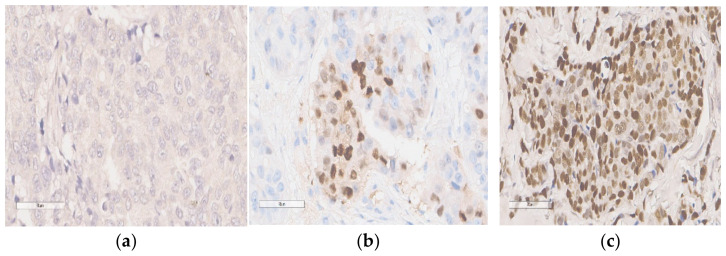
Representative immunohistochemical staining of p53 expression level. (all 400× magnification): (**a**) negative p53 expression; (**b**,**c**) positive p53 expression.

Sixty-seven pre-treatment paraffin blocks from every sixty-seven patients were compiled. Hematoxylin–eosin (HE) staining was first performed on one 4 μm section of each paraffin block and checked by two pathological anatomy experts to verify that the presentable number of carcinoma cells was present and the fixation quality was acceptable for immuno-histochemistry IHC analysis (Figure 1). See the Data Availability Statement for the full link to our HE staining protocol conducted in this study.

Expression of p53 was immunohistochemically evaluated (LSAB method) using a mouse monoclonal antibody to p53 (Clone DO7; Dako). All staining was carried out in a single lab: Hasan Sadikin Pathological Anatomy Laboratory in Bandung, West Java. See the Data Availability Statement for the full link to the IHC staining protocol for p53 expression conducted in this study.

Expression of p53 was scored by assigning the proportion of the stained nuclei of the cells; any intensity of nuclear staining in breast carcinoma epithelium counted for the positive proportion. The two pathological anatomy experts read a positive proportion of the tumor to avoid bias.

Ten percent of positive cell nuclei was considered positive for p53 expression and thus was a surrogate marker for p53 mutations in the patient’s tissue [16,19]. The method, cut-off, and antibody were explicitly chosen in conjunction with previous literature by Kikuchi et al. (2013), which stated that such arrangements have a significant association with clinicopathological features in luminal B HER2-negative patients [23].

### 2.3. Statistical Analysis

The sample size was calculated by taking power at 95%, and the confidence level was 95%. We calculated the sample using data from a previous study, in which the percentage of control subjects exposed was 0.88, and p53 positive expression is associated with relapse during endocrine therapy with an odds ratio of 4.82 [24]. We defined the ratio of the two analyzed groups as 1:1. We used the chi-squared test to investigate the relationships between the two groups (with and without primary endocrine therapy resistance) in terms of expression and clinicopathological characteristics for all patients included in the study. We calculated inter-observer agreement and kappa scores to observe discordance between the two observers. Furthermore, logistic regression analysis was applied to determine the likelihood of p53 becoming the predictor of predictive ET resistance compared with the other confounding variables. Statistical analysis is generated using Statistical Package for the Social Sciences (SPSS) for Windows 25.0.

## 3. Results

In this study, we analyzed the IHC expression of p53 and clinical data in 29 luminal B HER2-negative cases with primary ET resistance and 38 cases without primary ET. (See Data Availability Statement).

We excluded 309 patients, with the majority reason (179 patients—58.68% of the excluded population) being incomplete clinical data (pre-treatment and/or follow-up data). Other reasons for exclusion were diagnosed as a metastatic disease in the pre-treatment period (76 patients—24.60%), lost or poor quality of paraffin block (14 patients—4.53%), obesity (16 patients—5.17%), bilateral diseases (11 patients—3.60%), and unresponsive to neo-adjuvant chemotherapy and/or radiotherapy (six patients—1.94%).

The mean age of patients was 48.68 ± 9.43, with the youngest being 25 and the oldest 69 years old. All ER statuses of the patients were positive. Further, we carried out a bivariate analysis between p53 and primary ET resistance, as depicted in Table 2.

### Figures and Tables

Based on the statistical analysis results, we found no clinicopathological difference between the groups with and without primary ET resistance (Table 1). This means these two groups are equal in their clinicopathological characteristics and, therefore, comparable.

We found that positive p53 expression is significantly higher in patients with primary ET resistance (22 patients—73.33%) than without primary ET resistance (eight patients—26.67%), OR 11.78 (95% CI: 3.72—37.37, *p*-value < 0.0001). Two pathological anatomy experts interpreted these p53 expression results with an inter-observer agreement of 94.03% and a kappa-score of 0.88. (See Data Availability Statement for the link to full details of all patients’ immunohistochemical staining).

Due to insignificant results for all confounding variables in Table 1, we could not proceed to multivariate analysis.

## 4. Discussion

To our knowledge, this is the first study to observe p53 expression and its association with primary ET resistance in locally advanced luminal B HER2-negative BC.

Luminal B BC has several poor biological characteristics, such as a higher mutational load and a higher proliferation index (such as Ki-67), compared with luminal A BC. These features are prominent hallmarks in this group, thus making it more prone to ET resistance [8,25,26,27]. In such conditions, resistant cancer cells could proliferate and develop new cells with more biological mutations, eventually causing clinical jeopardy in patients [28,29]. These conditions could emerge in patients as new lesions in previously well-treated post-operative scars, newly found metastatic disease in follow-up, or progressive disease after several months in follow-up [10]. These will require re-operation or radiotherapy of the new lesion, escalation of chemotherapy, and often palliative treatment in late disease; all of these will result in poor prognosis and eventually increased mortality in the luminal B BC patients [7,10,30]. A study by Li et al. (2016) even noticed that such recurrence and metastasis risk had obviously decreased for non-luminal patients after a 2–5-year period of treatment, but for luminal B BC patients, the risks were still present during the same period [7].

Many studies and clinical trials have been conducted in the hope of finding the proper treatment for patients with ET resistance, but to no avail until this article is written [29,31]. The leading cause of this struggle is that mechanisms within the ET resistance are multifactorial and intricate [32,33,34]. However, the molecular mechanisms by which ER and PR are working to transcript their target genes are already comprehensible [14,35].

Our previous published review has noted that p53 roles are entangled in several prominent pathways involved in ER and PR molecular mechanisms, such as the NF-kB pathway, PI3K/Akt/mTOR pathway, and other ER target genes, thus making p53 mutations a plausible predictor for ET resistance, especially in the luminal B group [14]. p53 is a well-known tumor marker, and its expression is also versatile, as it could be tested readily in many laboratories [19,36]. However, to our knowledge, no research focuses predominantly on evaluating p53 expression in luminal B BC and its association with primary ET resistance, and this is the first study specifically designed for such a purpose.

For the p53 expression cut-off in IHC staining, we decided on 10% as a positive expression because it already correlates significantly with clinicopathological characteristics, specifically in the luminal B Her2-negative population [23]. With bivariate analysis (Table 2), we could evaluate that a group of luminal B HER2-negative patients with recurrent or progressive disease in the first two years of ET have significant p53 expression, OR 11.78 (95% CI: 3.72–37.37, *p*-value < 0.0001). This finding was consistent with other studies in the hormonal receptor-positive HER2-negative BC group [16,24,28].

A study by Yamashita et al. (2006) in 73 BC patients proved that p53 protein accumulation and high Ki67 expression are more resistant to aromatase inhibitors in metastatic disease patients (*p*-values of 0.0049 and 0.024, respectively) [24]. Another study by Yamamoto et al. (2014) also noted that positive p53 expression is associated with early recurrence in all clinical stages of postmenopausal HR+ BC (*p*-value < 0.0001) [37]. But these findings were also not specifically found for luminal B HER2 negative, as patients with HER2 positive and patients with Ki67 < 14% were included in their analysis [37]. It is well known that BC is highly heterogeneous, and HER2 positive expression and high Ki67 expression are related to overall BC recurrence; therefore, they could affect such results [38,39].

An extensive study by Anh et al. (2013) in 15,598 BC patients noted that positive p53 expression in hormonal receptor (HR)+/Her2-negative patients is significantly associated with the patient’s response to hormonal therapy and, therefore, could affect overall survival (OS) and breast cancer-specific survival (BCSS) [16]. Although this study is extensive with a much bigger sample than ours, it was not designed to specifically study p53 expression and its association with ET resistance (i.e., recurrence and progression within the 2-year ET period), but its association with OS. It is also not specifically conducted in locally advanced luminal B HER2-negative patients but in all BC patients at all stages, meaning it could have more bias in the result and interpretation [16].

It is also well known that IHC is affected by subjectivity. Anh et al. stated that their study is conducted retrospectively and just based on the BC registry (meaning it could be several pathologists who read the IHC analysis) and not centrally validated; therefore, it could have a bias in their result [16]. In this study, we provided two pathological anatomy experts who interpreted the p53 analysis expression to minimize this bias, and furthermore, we analyzed inter-observer agreement and the kappa score. We found inter-observer agreement of 94.03% and a kappa score of 0.88, which means the IHC result in our study is quite credible [40].

Those aforementioned three studies also did not homogenously collect the locally advanced BC patients, which could affect the study result as, e.g., the metastatic disease could have more significant mutations and more aggressive biological behavior and, therefore, could have a higher risk for recurrence than early BC. Besides metastatic disease, in an effort to minimize bias in our study, we also tried to exclude several risk factors that could affect ET resistance, such as obesity (BMI ≥ 30 kg/m^2^), bilateral breast cancer, and any unresponsiveness to neoadjuvant chemotherapy, beside metastatic disease [12,41,42,43].

Confounding variables in this study (patient’s age, stage, histological grade, chemotherapy, and radiotherapy usage) are not significantly different in both groups, and thus, we cannot proceed to multivariate analysis. But these findings could be due to our limited sample. For instance, patients with young BC (age < 35 years old at diagnosis) were only four, and patients admitted for radiotherapy were only six. Therefore, these findings should be explored more in future studies because they are inconsistent with previous research with a larger sample [12,16,43,44].

This study is distinct from other studies as we differentiate the ER expression into five categories: negative (<1%, 1–20%, 20–50%, 50–80%, and ≥80%). This is our effort to further investigate the difference in ER expression level, as many studies have discussed the gradation of ER expression level as an essential factor for endocrine therapy resistance [45]. A study by Sleightholm et al. stated that the percentage of ER positivity in BC provides additional prognostic value than a dichotomy (positive/negative) based on a 1% cut-off only. The American Society of Clinical Oncology (ASCO) Guideline 2020 for Estrogen and Progesterone Receptor Testing in Breast Cancer strongly suggests that low positivity of ER expression (1–10%) is associated with limited data on endocrine therapy benefits [46]. But overall, we did not find any significant difference in ER expression level between the case and control groups ( *p*-Value: 0.685).

This study has several limitations. As we mentioned before, we realized that we have a limited number of samples, and our study design was conducted in a retrospective and cross-sectional fashion due to the study’s feasibility. A prospective cohort design could provide a more accurate depiction of p53 association with primary ET resistance in luminal B HER2-negative patients. Therefore, we noted that the results and conclusions should be interpreted cautiously.

We acknowledged that patients included in our study used various ET drugs with diverse mechanisms of action; therefore, this caused bias. Undeniably, individual variability in drug efficacy will result in different outcomes. For instance, the inactivity of cytochrome p450 enzymes caused by gene polymorphism may cause relative resistance to Tamoxifen [47]. A more focused population selection study in one drug group (e.g., selective estrogen receptor modulator (SERM) users only) would address such a matter. However, we showed biological p53 expression differences since the pre-treatment period significantly affects ET resistance, regardless of the anti-endocrine drug type used.

Another limitation of our study was that we investigated p53 expression, ER expression, and PR expression using immuno-histochemistry (IHC) in a paraffin block. However, a more accurate result could be achieved by using genomic testing with fresh tissue [48]. Moreover, p53 expression detection using the IHC method has a probability of bias as p53 has many isoforms [49].

In addition, we only conducted a single p53 expression analysis during the pre-treatment period. It is known that BC in its locally advanced stage could undergo genetic changes throughout treatment and the disease course [43,50]. Thus, further studies with serial p53 mutation analysis during the patients’ treatment period will be beneficial. It could better understand its role in endocrine therapy resistance and generate more accurate timings to check the p53 mutation for overall prognostic usage.

Last but not least, we did not analyze several fundamental biomarkers in ET resistance, such as NF-kB and PIK3CA mutations. Loss of ET usage due to ET resistance drives clinicians and scientists to explore further alternative therapies for luminal BC with ET resistance [51]. Both biomarkers are also related to immunology and immunotherapy, as is their association with luminal BC [52,53,54]. Luminal B BC is also deemed more immunogenic than luminal A BC, expressing higher levels of inflammatory cytokines than luminal A BC. Therefore, nowadays, immunotherapy is an interesting topic to be explored in luminal BC with ET resistance [55,56,57]. Furthermore, recent studies noted that p53 mutations were found to promote higher immunogenic activity in BC, which means BC with p53 mutation could be more immunogenic, and immunotherapy could possibly be given in such patients [58,59]. Therefore, it is plausible that subsequent studies should explore more about these immunological markers, such as tumor-infiltrating lymphocytes (TILs), to see their association with p53 roles in ET resistance.

However, despite all the study limitations, we could show in this research that p53 protein expression as a surrogate marker for p53 mutations is significantly associated with primary ET resistance. As we mentioned above, this is the first study to do so, and we do believe this topic is important, relevant to the daily problems encountered by clinicians in locally advanced luminal B HER2-negative BC in Indonesia, and could be researched in the future with some of the considerations above.

## 5. Conclusions

p53 expression could be a beneficial marker for clinicians to determine which luminal B HER2-negative patients with locally advanced disease would likely have primary ET resistance, so they could receive more scrutinized monitoring to improve prognosis. However, this finding requires further studies with larger sample sizes using a prospective cohort design with patients who only use one type of ET drug. We also recommend serial analysis of genomic testing for p53 mutations using fresh specimens throughout patients’ treatment periods and analyzing their association with immunologic markers, especially TILs.

## Figures and Tables

**Table 1:** Clinicopathological characteristics.

Variable	Total Sample = 67		With Primary ET Resistance *n* = 29 (44%)	Without Primary ET Resistance *n* = 38 (56%)	*p*-Value
	n	%	n (%)	n (%)	
Age (year)					
<35	4	5.97	3 (75)	1 (25)	0.187
≥35	63	94.03	26 (41.27)	37 (58.73)	
ER status					
Negative (<1%)	0	0	0	0	0.685
1–20%	6	8.96	3 (50)	3 (50)	
20–50%	11	16.42	3 (27.27)	8 (72.73)	
50–80%	14	20.9	6 (42.86)	8 (57.14)	
≥80	36	53.73	17 (47.22)	19 (52.78)	
Progesterone Receptor Status					
Positive	63	94.03	26 (41.27)	37 (58.73)	0.187
Negative	4	5.97	3 (75)	1 (25)	
Stage					
Stage IIIa	26	40	14 (53.85)	12 (46.15)	0.152
Stage IIIb	38	58.46	13 (34.21)	25 (65.79)	
Stage IIIc	1	1.54	1 (100)	0 (0)	
Histopathological Grade					
I	1	1,49	0 (0)	1 (100)	0.265
II	29	43.28	10 (34.48)	19 (65.52)	
III	37	55.22	19 (51.35)	18 (48.65)	
Neo-adjuvant chemotherapy					
Taxane based	10	14.93	5 (50)	5 (50)	0.061
Doxorubicin based	47	70.15	16 (34.04)	31 (65.96)	
Taxan and doxorubicin combination	6	8.96	5 (83.33)	1 (16.67)	
Taxan and platinum combination	4	5.97	3 (75)	1 (25)	
Radiotherapy					
Yes	7	10.45	4 (57.14)	3 (42.86)	0.457
No	60	89.55	25 (41.67)	35 (58.33)	
Endocrine Therapy					
SERM	26	38.81	7 (26.92)	19 (73.08)	0.296
Aromatase inhibitor	19	28.36	10 (52.63)	9 (47.37)	
SERD	1	1.49	1 (100)	0 (0)	
SERM, aromatase inhibitor	5	7.46	2 (40)	3 (60)	
LHRH/GnRH agonist, SERM	16	22.88	9 (56.25)	7 (43.75)	

ET: endocrine therapy, SERM: selective estrogen receptor modulator, AI: aromatase inhibitor, SERD: selective estrogen receptor degrader, LHRH: luteinizing hormone-releasing hormone, GnRH: gonadotropin-releasing hormone.

**Table 2:** p53 expression level group with and without primary ET resistance.

Variable	Total Sample = 67	With Primary ET Resistance *n* = 29 (44%)	Without Primary ET Resistance *n* = 38 (56%)	*p*-Value	OR	95% CI
	*n* (%)	*n* (%)	*n* (%)			
p53 expression						
Positive expression	37 (55.22)	22 (73.33)	8 (26.67)	<0.0001	11.78	3.72–37.37
Negative expression	30 (45.45)	7 (18.92)	30 (81.08)			

ET: endocrine therapy, OR: odds ratio, CI: confidence interval.

## Data Availability

Link to Full details of all de-identified patient data: https://doi.org/10.12688/f1000research.129906.1, accessed on 8 May 2023. Link to the full protocol of the HE staining procedure https://www.protocols.io/view/hematoxyllin-eosin-he-staining-4r3l27zo3g1y/v1, accessed on 8 May 2023. Link to the full protocol of our Immunohistochemistry staining procedure https://www.protocols.io/view/immunohistochemistry-for-p53-staining-in-breast-ca-5qpvord7dv4o/v1, accessed on 8 May 2023. Link to full details of all 67 patients’ immunohistochemical staining: https://doi.org/10.12688/f1000research.129906.1, accessed on 8 May 2023 Link to Representative Figures for ER Expression Level: https://doi.org/10.12688/f1000research.129906.1, accessed on 8 May 2023.
